# Online Public Attention During the Early Days of the COVID-19 Pandemic: Infoveillance Study Based on Baidu Index

**DOI:** 10.2196/23098

**Published:** 2020-10-22

**Authors:** Xue Gong, Yangyang Han, Mengchi Hou, Rui Guo

**Affiliations:** 1 School of Public Health Capital Medical University Beijing China

**Keywords:** Baidu Index, public attention, time lag cross-correlation analysis, COVID-19

## Abstract

**Background:**

The COVID-19 pandemic has become a global public health event, attracting worldwide attention. As a tool to monitor public awareness, internet search engines have been widely used in public health emergencies.

**Objective:**

This study aims to use online search data (Baidu Index) to monitor the public’s attention and verify internet search engines’ function in public attention monitoring of public health emergencies.

**Methods:**

We collected the Baidu Index and the case monitoring data from January 20, 2020, to April 20, 2020. We combined the Baidu Index of keywords related to COVID-19 to describe the public attention’s temporal trend and spatial distribution, and conducted the time lag cross-correlation analysis.

**Results:**

The Baidu Index temporal trend indicated that the changes of the Baidu Index had a clear correspondence with the development time node of the pandemic. The Baidu Index spatial distribution showed that in the regions of central and eastern China, with denser populations, larger internet user bases, and higher economic development levels, the public was more concerned about COVID-19. In addition, the Baidu Index was significantly correlated with six case indicators of new confirmed cases, new death cases, new cured discharge cases, cumulative confirmed cases, cumulative death cases, and cumulative cured discharge cases. Moreover, the Baidu Index was 0-4 days earlier than new confirmed and new death cases, and about 20 days earlier than new cured and discharged cases while 3-5 days later than the change of cumulative cases.

**Conclusions:**

The national public’s demand for epidemic information is urgent regardless of whether it is located in the hardest hit area. The public was more sensitive to the daily new case data that represents the progress of the epidemic, but the public’s attention to the epidemic situation in other areas may lag behind. We could set the Baidu Index as the sentinel and the database in the online infoveillance system for infectious disease and public health emergencies. According to the monitoring data, the government needs to prevent and control the possible outbreak in advance and communicate the risks to the public so as to ensure the physical and psychological health of the public in the epidemic.

## Introduction

As a Public Health Emergency of International Concern [1], the COVID-19 pandemic swept 215 countries and regions with high transmission speed, wide infection range, and difficulty in prevention and control [2,3]. As of July 21, 2020, the number of cumulative confirmed cases worldwide exceeded 14.7 million, and the number of cumulative death cases exceeded 600,000 [3]. COVID-19 has caused challenges and threats to public health in China and the world, attracting widespread public attention.

Existing online search data is a voluntary expression of the public, reflecting public attention and needs; compared with the traditional survey data, it has greater timeliness, objectivity, and credibility [4-7]. Search engines, as the representative of internet-based sources, have been proven to detect the initial evidence of an epidemic [8]. Internet-based technology provides essential benefits for improving the transparency of epidemic reporting and complementing the traditional surveillance, enabling health institutions to respond quickly in a targeted manner, thereby reducing morbidity, mortality, and disease outbreaks [8-11]. Many studies have shown that the internet search engine, as a monitoring platform for public concern in public health emergencies, has become an “outpost” for early warning of the epidemic. On the one hand, the internet search data correlated with traditional reported data (such as laboratory confirmed data and death data) [12,13]. On the other hand, internet search data tended to be ahead of case data. For example, Polgreen et al [14] used the Yahoo search engine to collect influenza data and found that it was 1-3 weeks and 5 weeks earlier than the routine reporting of laboratory confirmed cases and influenza deaths, respectively; Ginsberg et al [15] proposed the concept of Google Flu Trends in 2009 and found that Google predicted results were 1-2 weeks earlier than the Centers for Disease Control and Prevention flu surveillance system report in the United States. For COVID-19, Li et al [16] found that the internet search data from Google Trends, Baidu Index (BDI), and Sina Weibo Index was 8-10 days earlier for new laboratory-confirmed cases and 5-7 days earlier for new suspected cases [16]. Therefore, it is an important reference for social demand monitoring. In China, Baidu search’s penetration rate reached 90.9% among internet search engine users as of October 2019, equivalent to Google’s role in western countries [17]. At present, scholars have used Google and Baidu search to obtain internet data. Their applicability in monitoring public attention of public health emergencies such as influenza [15-18], H7N9 [19,20], and Dengue [21,22] has been widely confirmed.

To date, none of the studies combine temporal and spatial relationship, and relevance between search engines and public attention under the COVID-19 pandemic and focus on the differences in public concerns between hardest hit and non–hardest-hit areas. Thus, this study aims to use BDI to monitor the public’s attention and verify internet search engines’ function in public attention monitoring of public health emergencies.

## Methods

### Real-World Databases

We selected six case indicators for real-world data, including *new confirmed cases*, *new death cases*, *new cured discharge cases*, *cumulative confirmed cases*, *cumulative death cases*, and *cumulative cured discharge cases* (Multimedia Appendix 1). We collected real-world data from January 20, 2020, to April 20, 2020. The National Health Commission of the People’s Republic of China has compiled and released the number of cases in each province daily from January 20, 2020. Considering the data reliability, we used the official data reported by the government, so we selected January 20 as the start time of the study. The reason for choosing April 20, 2020, as the deadline is that Hubei Province, the hardest hit area, reported only three new confirmed cases in the past month. Medical teams from other provinces had been withdrawn one after another from Hubei Province. The national economic and social order is gradually recovering. It can be considered that the COVID-19 epidemic in China had entered the normalization stage. Real-world data comes from the daily outbreak notification of the official website of the National Health Commission of the People’s Republic of China [23].

### BDI Databases

In the early stage of the epidemic, there was no unified name for COVID-19. Taking into account the BDI algorithm, a common expression of the Chinese public, and scholars’ research on the main topics discussed by netizens during the epidemic [22], we selected “Novel coronavirus (新型冠状病毒),” “Pneumonia (肺炎),” “New pneumonia (新型肺炎),” “Novel Coronavirus Pneumonia (新型冠状病毒肺炎),” “Epidemic (疫情),” “Wuhan (武汉),” and “Wuhan Pneumonia (武汉肺炎),” seven Chinese words with large data values as the BDI-related keywords. These keywords include pneumonia, Wuhan, virus, and other words that can represent epidemic events. The combined BDI of seven keywords was used as the BDI data for COVID-19. We collected BDI data from December 8, 2019, to April 20, 2020, which is different from the real-world data collection time because BDI was already high on January 20, 2020. To more fully demonstrate the changes in public attention in the early stage of the epidemic, the start time of BDI data collection was advanced to the onset of the first confirmed COVID-19 case notified by the Wuhan Municipal Health Commission. BDI data comes from the BDI official website [24].

### Analysis

We used Excel 2019 (Microsoft Corporation) for database construction. The curve of COVID-19 case-related indicators and BDI was plotted to describe the development trend of the epidemic and the changing trend of public attention. Time lag cross-correlation analysis of BDI and case data was performed using SPSS 26.0 English version (IBM Corp) to explore the correlation between public concern and the actual epidemic. Considering the data comparability, the correlation analysis time was from January 20, 2020, to April 20, 2020.

## Results

### COVID-19 Epidemic Trend

**Figure 1:**
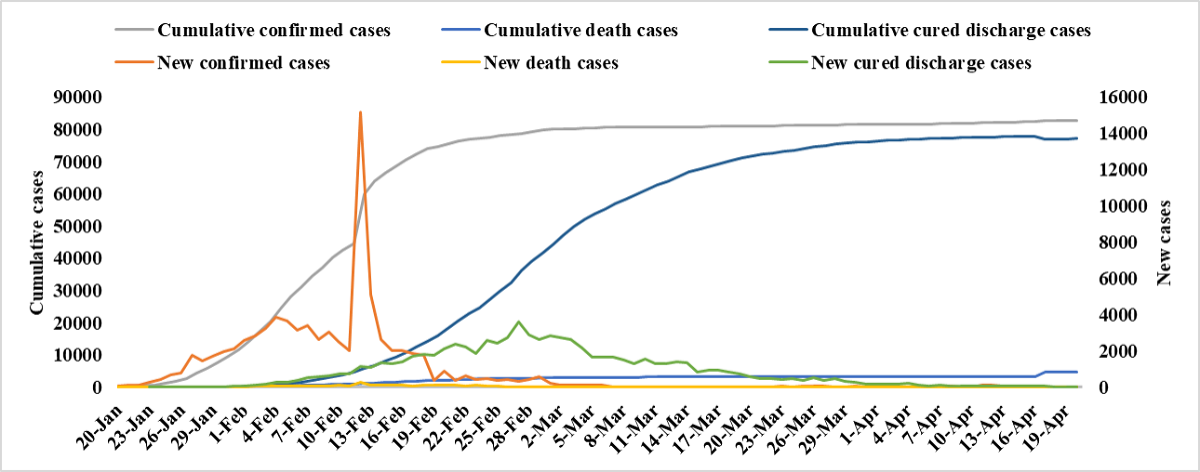
The epidemic characteristics of COVID-19 in China from January 20 to April 20, 2020.

We used six case indicators from real-world data to depict the characteristics of the COVID-19 outbreak in China between January 20, 2020, and April 20, 2020 (Figure 1).

### BDI Temporal Trend

**Figure 2:**
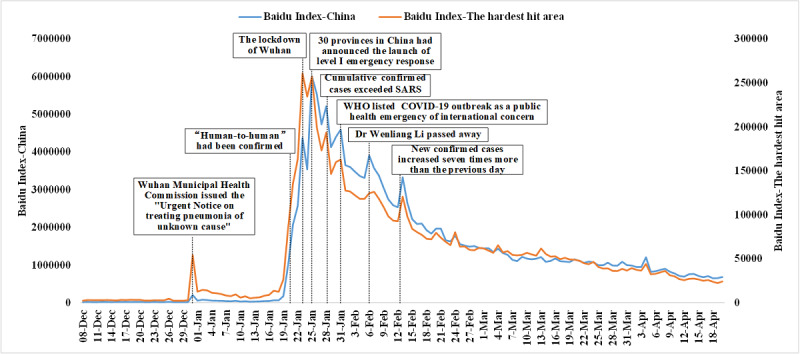
The changing trend of the Baidu Index of COVID-19 in China from January 2 to April 20, 2020. WHO: World Health Organization.

During December 8, 2019, to April 20, 2020, the trend of the COVID-19 BDI in China experienced a state of “developing from nothing, reaching a peak, fulling volatility, and stabilizing gradually” (Figure 2). At the beginning of the observation period, the BDI was at an extremely low level. The first small peak appeared on December 31, 2019. The BDI increased significantly from January 20, with a small peak on January 23, and the BDI reached its peak on January 25. Subsequently, the BDI fluctuated and declined, during which there were several small peaks on January 28, January 31, February 6, and February 13. After February 13, the BDI declined steadily with the decrease of new confirmed cases and the increase of new cured and discharged cases. By the end of observation (April 20, 2020), the BDI was still significantly higher than the level at the beginning of observation (December 8, 2019).

### BDI Spatial Distribution

**Figure 3:**
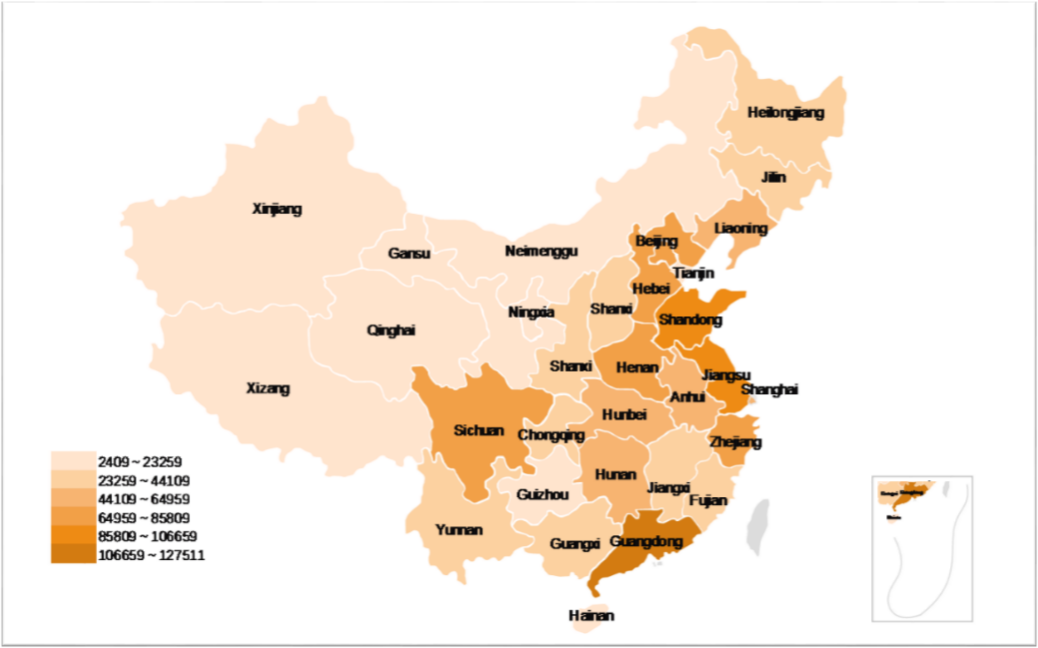
The spatial distribution of daily average Baidu Index from December 8, 2019, to April 20, 2020.

Figure 3 and Table 1 show the daily average BDI and per capita BDI of all provinces in China from December 8, 2019, to April 20, 2020. The BDI was significantly concentrated in the central and eastern regions of China. The daily average BDI in Guangdong, Shandong, Jiangsu, Beijing, Hebei, Zhejiang, Sichuan, Henan, Hubei, and Liaoning exceeded 50,000 in search frequency. Taking into account the different population densities and internet user bases in different provinces, we calculated the per capita BDI of each province through internet users (per capita BDI = daily average BDI / internet users). The top ten provinces were Beijing, Tianjin, Shanghai, Liaoning, Hubei, Jilin, Shandong, Hebei, Heilongjiang, and Jiangsu.

**Table 1:** The daily average BDI and per capita BDI in each province from December 8, 2019, to April 20, 2020.

Provinces	Daily average BDI^a^	Internet users × 10,000	Per capita BDI/10,000
Guangdong	127,511	14,106.9	9.04
Shandong	96,490	8418.6	11.46
Jiangsu	89,553	7979.5	11.22
Beijing	74,987	3291.1	22.78
Hebei	74,383	6505.3	11.43
Zhejiang	73,434	6833.6	10.75
Sichuan	69,057	7332.0	9.42
Henan	67,697	7766.5	8.72
Hubei	53,996	4552.0	11.86
Liaoning	51,363	3905.9	13.15
Shanghai	49,003	3032.0	16.16
Anhui	47,715	4596.3	10.38
Hunan	44,501	5226.6	8.51
Fujian	36,810	3793.6	9.70
Jiangxi	33,595	3340.8	10.06
Shanxi	33,011	3726.8	8.86
Heilongjiang	33,000	2894.9	11.40
Shanxi	32,243	3086.3	10.45
Chongqing	28,544	2862.2	9.97
Jilin	27,191	2366.2	11.49
Guangxi	26,405	4130.8	6.39
Yunnan	24,577	3918.9	6.27
Tianjin	23,079	1352.5	17.06
Neimenggu	22,845	2508.1	9.11
Guizhou	21,656	3323.1	6.52
Gansu	17,628	2210.6	7.97
Xinjiang	14,231	1992.5	7.14
Hainan	9759	914.0	10.68
Ningxia	6015	694.6	8.66
Qinghai	5453	559.0	9.75
Xizang	2409	260.2	9.26

^a^BDI: Baidu Index.

### Time Lag Cross-Correlation Analysis

#### Correlation Analysis of BDI and Real-World Data in China

We conducted the time lag cross-correlation analysis between national BDI and six case indicators to explore key case indicators that may cause public attention fluctuations and the relationship between the BDI and case indicators. The results showed that, except for new cured and discharged cases, the other five case indicators were significantly correlated with BDI within the time range of 6 days earlier or lagging (Table 2). The correlation between the BDI and new confirmed cases was the highest at a lag of 0 days (Spearman correlation coefficient 0.795). The correlation with new death cases was highest at a lag of –4 days (Spearman correlation coefficient 0.876). The correlation between the BDI and cumulative confirmed cases, cumulative death cases, and cumulative cured and discharged cases reached the highest level at a lag of 5 days, 4 days, and 3 days, respectively (Spearman correlation coefficients were 0.989, 0.983, and 0.947, respectively). That is, the public attention to the epidemic was 4 days earlier than the change of new death cases and 3-5 days later than the change of cumulative cases.

**Table 2:** The correlation between the national COVID-19 Baidu Index and real-world data from January 20 to April 20, 2020.

Baidu Index		New confirmed cases	New death cases	New cured discharge cases	Cumulative confirmed cases	Cumulative death cases	Cumulative cured discharge cases
**Lag –6 days**							
	Spearman correlation coefficient	0.689	0.868	0.452	–0.576	–0.583	–0.678
	*P* value	<.001	<.001	<.001	<.001	<.001	<.001
**Lag –5 days**							
	Spearman correlation coefficient	0.721	0.873	0.391	–0.633	–0.639	–0.729
	*P* value	<.001	<.001	<.001	<.001	<.001	<.001
**Lag –4 days**							
	Spearman correlation coefficient	0.751	0.876	0.325	–0.692	–0.695	–0.793
	*P* value	<.001	<.001	.002	<.001	<.001	<.001
**Lag –3 days**							
	Spearman correlation coefficient	0.775	0.865	0.255	–0.753	–0.754	–0.846
	*P* value	<.001	<.001	.02	<.001	<.001	<.001
**Lag –2 days**							
	Spearman correlation coefficient	0.776	0.855	0.193	–0.814	–0.814	–0.905
	*P* value	<.001	<.001	.07	<.001	<.001	<.001
**Lag –1 day**							
	Spearman correlation coefficient	0.789	0.841	0.180	–0.878	–0.875	–0.933
	*P* value	<.001	<.001	.09	<.001	<.001	<.001
**Lag 0**							
	Spearman correlation coefficient	0.795	0.806	0.170	–0.942	–0.941	–0.942
	*P* value	<.001	<.001	.11	<.001	<.001	<.001
**Lag 1 day**							
	Spearman correlation coefficient	0.780	0.769	0.165	–0.977	–0.974	–0.941
	*P* value	<.001	<.001	.12	<.001	<.001	<.001
**Lag 2 days**							
	Spearman correlation coefficient	0.772	0.748	0.165	–0.983	–0.978	–0.942
	*P* value	<.001	<.001	.12	<.001	<.001	<.001
**Lag 3 days**							
	Spearman correlation coefficient	0.759	0.738	0.165	–0.987	–0.982	–0.947
	*P* value	<.001	<.001	.12	<.001	<.001	<.001
**Lag 4 days**							
	Spearman correlation coefficient	0.759	0.733	0.162	–0.988	–0.983	–0.945
	*P* value	<.001	<.001	.13	<.001	<.001	<.001
**Lag 5 days**							
	Spearman correlation coefficient	0.763	0.723	0.163	–0.989	–0.983	–0.944
	*P* value	<.001	<.001	.13	<.001	<.001	<.001
**Lag 6 days**							
	Spearman correlation coefficient	0.754	0.729	0.164	–0.989	–0.983	–0.943
	*P* value	<.001	<.001	.13	<.001	<.001	<.001

**Figure 4:**
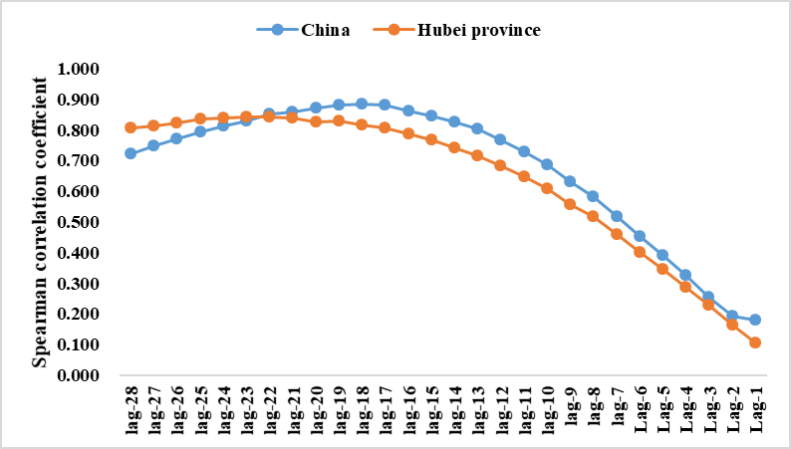
The correlation between Baidu Index and new cured and discharged cases in Hubei province and China.

To further explore the relationship between the BDI and new cured and discharged cases, we analyzed the correlation between the two within the 4 weeks lagging. The results showed that the correlation between the BDI and new cured and discharged cases was the highest at a lag of –18 days (Spearman correlation coefficient 0.883). That is, public attention to the epidemic was 18 days earlier than the change of new cured and discharged cases. The change of the Spearman correlation coefficient is shown in Figure 4.

#### Correlation Analysis of BDI and Real-World Data in Hubei Province

To understand whether the public attention to the epidemic in Hubei Province, the hardest hit area, is different from that of the whole country, we conducted the time lag cross-correlation analysis between the BDI and six case indicators of Hubei Province. The results were consistent with the correlation analysis of the national BDI and case data (Table 3). The correlation between the BDI and new confirmed cases was the highest at a lag of –1 day (Spearman correlation coefficient 0.870), and the correlation with new death cases was highest at a lag of –4 days (Spearman correlation coefficient 0.853). The correlation between BDI and cumulative confirmed cases, cumulative death cases, and cumulative cured and discharged cases reached the highest level at a lag of 2 days, 2 days, and 3 days, respectively (Spearman correlation coefficients were 0.992, 0.993, and 0.985, respectively). That is, the public attention to the epidemic was 1 day and 4 days earlier than the change of new confirmed cases and new death cases, and 3-5 days later than the change of three cumulative cases.

**Table 3:** The correlation between COVID-19 Baidu Index and real-world data from January 20 to April 20, 2020, in Hubei Province.

Baidu Index in hardest hit area		New confirmed cases	New death cases	New cured discharge cases	Cumulative confirmed cases	Cumulative death cases	Cumulative cured discharge cases
**Lag –6 days**							
	Spearman correlation coefficient	0.819	0.852	0.401	–0.620	–0.622	–0.613
	*P* value	<.001	<.001	<.001	<.001	<.001	<.001
**Lag –5 days**							
	Spearman correlation coefficient	0.839	0.850	0.346	–0.678	–0.681	–0.672
	*P* value	<.001	<.001	<.001	<.001	<.001	<.001
**Lag –4 days**							
	Spearman correlation coefficient	0.858	0.853	0.287	–0.737	–0.741	–0.733
	*P* value	<.001	<.001	.006	<.001	<.001	<.001
**Lag –3 days**							
	Spearman correlation coefficient	0.862	0.837	0.228	–0.799	–0.803	–0.794
	*P* value	<.001	<.001	.03	<.001	<.001	<.001
**Lag –2 days**							
	Spearman correlation coefficient	0.866	0.824	0.164	–0.862	–0.866	–0.857
	*P* value	<.001	<.001	.12	<.001	<.001	<.001
**Lag –1 day**							
	Spearman correlation coefficient	0.870	0.796	0.106	–0.927	–0.931	–0.920
	*P* value	<.001	<.001	.31	<.001	<.001	<.001
**Lag 0 days**							
	Spearman correlation coefficient	0.868	0.755	0.046	–0.984	–0.987	–0.978
	*P* value	<.001	<.001	.67	<.001	<.001	<.001
**Lag 1 days**							
	Spearman correlation coefficient	0.862	0.740	0.032	–0.989	–0.992	–0.983
	*P* value	<.001	<.001	.76	<.001	<.001	<.001
**Lag 2 days**							
	Spearman correlation coefficient	0.852	0.730	0.031	–0.992	–0.993	–0.984
	*P* value	<.001	<.001	.77	<.001	<.001	<.001
**Lag 3 days**							
	Spearman correlation coefficient	0.850	0.731	0.034	–0.991	–0.993	–0.985
	*P* value	<.001	<.001	.75	<.001	<.001	<.001
**Lag 4 days**							
	Spearman correlation coefficient	0.858	0.729	0.032	–0.991	–0.993	–0.985
	*P* value	<.001	<.001	.76	<.001	<.001	<.001
**Lag 5 days**							
	Spearman correlation coefficient	0.850	0.726	0.032	–0.992	–0.993	–0.984
	*P* value	<.001	<.001	.76	<.001	<.001	<.001
**Lag 6 days**							
	Spearman correlation coefficient	0.851	0.731	0.036	–0.992	–0.993	–0.984
	*P* value	<.001	<.001	.73	<.001	<.001	<.001

To further explore the relationship between the BDI and new cured and discharged cases in Hubei Province, we analyzed the correlation between the two within the time range of 4 weeks lagging. The results showed that the correlation between the BDI and new cured and discharged cases in Hubei Province was the highest at a lag of –22 days (Spearman correlation coefficient 0.844). That is, public attention to the epidemic was 22 days earlier than the change of new cured and discharged cases. The change of the Spearman correlation coefficient is shown in Figure 4.

## Discussion

### Principal Finding

The BDI can well fit the development of the COVID-19 epidemic in time and space, and we found that there were significant advance and delay effects between the BDI and the real-world case data. In brief, internet search data can be used for monitoring and early warning of public health emergencies, including the BDI used in this study and Google Trends used in other related studies. Thus, it can be said that internet search data supplements the government statistics official data lag [8].

### The Space-Time Distribution of the BDI Showed the Progress of the Epidemic

The BDI temporal trend showed that the changes in the BDI corresponded to COVID-19 news and major events, similar to other studies [25]. On December 30, 2019, Wuhan Municipal Health Commission issued the “Urgent Notice on treating pneumonia of unknown cause.” On this day, the expert team of the National Health Commission of the People’s Republic of China arrived in Wuhan to formally intervene in the investigation, which was the reason for the first small wave of the BDI. On January 20, 2020, expert Zhong Nanshan clarified the characteristics of COVID-19 person-to-person transmission. Simultaneously, the State Council incorporated COVID-19 into the Infectious Disease Law, which aroused wide public concern, and the BDI increased significantly. The BDI reached another small peak on January 23, probably due to the public panic caused by Wuhan’s lockdown on that day. On January 25, the BDI peaked as 30 provinces in China had announced the launch of a level I emergency response to public health emergencies. Subsequently, the BDI declined with fluctuation and several small peaks. The epidemic development events leading to the BDI fluctuations are marked in Figure 2.

Although internet search engines are reliable tools for epidemic infoveillance, information disseminated through the news media may affect search volumes and have an event amplification effect [26,27], thus increasing people’s attention to the epidemic. In this study, major events marked in Figure 2, which may lead to the BDI peak or small peak, had been widely reported by various social media. The reports quickly ignited the public’s attention to the epidemic, which led to an increase in BDI searches. In the early stage of the epidemic, the public knew little about the epidemic, and the information on social media was relatively fragmented. Besides, the epidemic was unstable and highly contagious, so the public actively searched for information to learn more about the epidemic. With the development of the epidemic and the accumulation of historical information, real-time dynamic information that can reflect the epidemic is also embedded in netizens’ social software. Users can directly read and obtain the information without an active search. Therefore, the public is more accustomed to passively accepting information, reducing its search behavior for epidemics [28].

The spatial distribution of the daily average BDI showed that the public attention to COVID-19 was concentrated in areas with denser populations, larger internet user bases, and higher economic development levels. In addition, there was a significant difference between the east and the west, with coastal provinces paying more attention to the epidemic than inland provinces. This finding was consistent with the research results of Han et al [25] and Sun et al [28]. As the epicenter of the COVID-19 outbreak, Wuhan, Hubei is undoubtedly a hot spot of public concern [25]. Most other regions such as the Beijing-Tianjin-Hebei and Guangdong, Jiangsu, Shandong, Zhejiang, and other coastal areas have denser populations, larger internet user bases, and higher economic development levels. In 2018, the top 10 provinces with daily average BDI accounted for 52.4%, 57.4%, and 59.1% of the 31 provinces in the country in terms of year-end population, GDP, and internet users, respectively [29]. In addition, the high levels of economic development and population density mean that these areas have convenient transportation infrastructure and network communications. These factors may increase the possibility of a faster epidemic spread and panic among the population, making it more challenging to prevent and control the epidemic. At the same time, we should also pay attention to areas with low internet search volume, as their information depends on passive access rather than active access. The government and other official propaganda media should broaden their channels so that people can obtain positive and effective information, and information fairness can be realized.

### BDI Has Significant Temporal Difference With Real-World Data

The time lag cross-correlation analysis showed a significant correlation between the BDI and the six case indicators regardless of the hardest hit area or other areas. In addition, the BDI had an advance effect compared to new cases and a lag effect compared to cumulative cases, indicating that the public was more sensitive to new cases. New cases can represent the severity of the epidemic. The public usually judges the current or future trend of the epidemic based on new cases. For confirmed, death, and cured and discharged cases, the cumulative cases are simply a superposition of new cases, representing the epidemic’s situation over a period of time.

In addition, in the correlation analysis between the BDI and new cases, the BDI was 0-4 days earlier than new confirmed and new death cases, and about 20 days earlier than new cured and discharged cases. In fact, “suspected- confirmed- treated- cured” is a phased development process with time sequence [28]. In the early stage of the outbreak, confirmed cases kept increasing, while the substantial increase in cured and discharged cases was later. Compared to new cases, the advance effect may represent a reporting bias rooted in testing delays [27]. COVID-19 is a newly discovered disease that requires laboratory testing for diagnosis. There is a time interval between the appearance of disease symptoms and the final diagnosis. The reporting bias further demonstrates the importance of real-time disease development assessment [16].

In addition, the BDI positively correlated with new cases but negatively correlated with cumulative cases. The positive correlation reflected the public’s panic about the epidemic. The public had little knowledge about the virus, so they paid particular attention to the daily new case data representing the epidemic’s progress. The negative correlation reflected the positive attitude of the Chinese public to the epidemic. With the continuous strengthening of epidemic prevention and control, the increase in cumulative confirmed cases and cumulative death cases had slowed down. The cumulative cured and discharged cases represented a positive trend of the epidemic. The larger the indicator value, the less the public will panic about the epidemic.

Previous studies mainly used daily new cases for time-delay correlation [16,22,27-33], while this study used new cases and confirmed cases. For confirmed, death, and cured discharge cases, new variables are the number of cases that increased in 1 day, and cumulative variables are the superposition of all the case data up to that day. The previously mentioned analysis showed that public attention was more consistent with new cases, which should be paid more attention to. Therefore, the content of epidemic information release should focus on the disclosure and interpretation of new cases and provide necessary explanations for possible causes of indicator disturbances so as to guide the public to correct risk perceptions and eliminate public panic. For example, the surge in new confirmed cases on February 12, 2020, was due to a change in statistical standards, with “clinically diagnosed cases” in Hubei included in the statistics of confirmed cases.

The early effect compared to new cases suggested that the BDI can be used as a sentinel in online infoveillance systems for infectious diseases and public health emergencies. The correlation analysis used in this study is the most common form of data monitoring, which is regularly applied to determine the relationship between internet-based and real-world data [8]. Gu et al [30] found that the erythromelalgia epidemic search index showed the uptrend about a week ahead of the official report because of the delayed reports from the local Center for Disease Control and Prevention. Future research can establish relevant models, including the vector autoregressive model, to predict the future trend of the epidemic based on past values of the real-world data [22,31].

### Similarities and Differences of Public Attention Between the Hardest Hit Area and Other Areas

The hardest hit area was consistent with the whole country in the trend of BDI or the time lag cross-correlation analysis, except the peak of public attention across the country laid behind the hardest hit area by 2 days. That means that in the early stage of the COVID-19 pandemic, the public in the hardest hit area were more sensitive and alert to online epidemic information, while the public in other areas may have a lag in the attention to the epidemic. Besides, from the correlation between the BDI and real-world data, the national correlation coefficient was generally higher than that of the hardest hit area, indicating that the national public paid more attention to real-world data than the hardest hit area. In the spatial distribution of the BDI, the daily average BDI and per capita BDI of Hubei Province were not the highest, which also proved this point. That is to say, although the national public attention laid behind the hardest hit area, the level of attention was high. A possible reason for the delayed access to information is that when we focus on something, we will search for more of it. In other words, the public need for awareness is the internal motivation for people to use information systems to obtain needed information. In addition, the public will be concerned later than people in the “epicenter” of the epidemic because of the delay in getting information, such as untimely, incomplete information disclosure, or distortion of information transmission. When the public in other areas suspect that they have insufficient or asymmetric information, the truth’s ambiguity will drive them to actively search for information to master sufficient COVID-19 knowledge [34], thus showing that the level of public attention is higher than that in the hardest hit area.

### Conclusions

In this study, we found that the public searched for COVID-19 information showing the epidemic’s progress. Moreover, people in the hardest hit area, with denser populations, larger internet user bases, and higher economic development levels, paid more attention to the epidemic’s development. We could set the BDI as the sentinel in the online infoveillance system for infectious disease and public health emergencies. The change of the BDI was significantly earlier than the new cases, especially the new cured and discharged cases, which means the government needs to prevent and control the possible outbreak in advance according to the monitoring data and communicate the risks to the public so as to ensure the physical and psychological health of the public in the epidemic.

### Limitations

This study has several limitations. First, this study only focused on the attention of Baidu search engine users to COVID-19. It did not consider public attention in other search engines or social media such as Sina Weibo and WeChat, which can only reflect part of the public’s attention to COVID-19. Second, since there was no uniform name for COVID-19 in the early stage of the epidemic, the public had a wide range of search terms. We only selected seven representative keywords to collect the BDI. Third, the Baidu search volume may be influenced by the media. Higgins et al [27] even pointed out that Google Trends and BDI may have better reliability in defining the epidemiology for common diseases with minor media coverage or rare diseases and conditions with higher audiences. Fourth, this study cannot avoid the influence of searchers’ age, occupation, and other demographic information. The average age of patients with COVID-19 is 51 years and nearly 80% of them are aged 30-69 years [35], but 90.2% of internet search engine users are younger than 50 years [36]. Moreover, people with different jobs may have different online search purposes and volumes. For example, experts, students, and doctors may conduct many searches due to work demands. However, due to Baidu’s privacy protection policy, other demographic information such as the occupation of searchers is not provided, so we could not obtain more user-related information [37].

## Appendix

Multimedia Appendix 1 Indicators and situations of COVID-19 cases and China epidemic area division.

## Abbreviations

BDI: Baidu Index
