# Supplementary material for: Online Public Attention During the Early Days of the COVID-19 Pandemic: Infoveillance Study Based on Baidu Index
Source: JMIR Public Health Surveill. 2020 Oct 22;6(4):e23098. doi: 10.2196/23098 (PMC7584450; doi:10.2196/23098)
Supplement: Multimedia Appendix 1 [file publichealth_v6i4e23098_app1.docx]

Appendix 1

| Table 1 Indicators and situations of COVID - 19 cases ^a^ | | | |
| --- | --- | --- | --- |
|  | Case Indicator | Time range | The clinical situation |
| 1 | new confirmed cases | per day | Laboratory test positive |
| 2 | new death cases | per day | confirmed death |
| 3 | new cured discharge cases | per day | Laboratory test negative |
| 4 | cumulative confirmed cases | total days | Laboratory test positive |
| 5 | cumulative death cases | total days | confirmed death |
| 6 | cumulative cured discharge cases | total days | Laboratory test negative |

^a^ Form made by author, according to the disclosure of case indicators in COVID-19 prevention and control: epidemic notification by National Health Commission of the People’ s Republic of China. <http://www.nhc.gov.cn/xcs/yqtb/list_gzbd.shtml>

| Table 2 China epidemic area division | | |
| --- | --- | --- |
|  | Type | Range |
| 1 | the hardest-hit area | Hubei province where the first case emerged in China, where has 68128 cumulative confirmed cases and 4512 cumulative death cases as of April 20, 2020.^a^ |
| 2 | other areas | Provinces in mainland China except the hardest-hit area. |

^a^ Form made by author, according to COVID-19 prevention and control: epidemic notification in April 20, 2020 by National Health Commission of the People’ s Republic of China. <http://www.nhc.gov.cn/xcs/yqtb/202004/b504a02486834baf8ed8149701a4175b.shtml>
